# Nucleoporins but not septins at the transition zone of cilia in *Paramecium *and *Tetrahymena*

**DOI:** 10.1186/2046-2530-4-S1-P28

**Published:** 2015-07-13

**Authors:** M Dziadosz, J Nowak, E Joachimiak, A Aubusson-Fleury, D Włoga

**Affiliations:** 1 The Nenki Institute of Exp. Biol., Dept Cell Biology, Polish Academy of Sciences, Warsaw, Poland; 2 CGM UPR3404, CNRS, Gif Sur Yvette, France

## 

The transition zone (TZ) of locomotory cilia participates in docking young basal bodies to the surface, stabilizes the basal body-membrane connection, forms a diffusion barrier and modulates the intraciliary transport. In vertebrate ciliated cells, nucleoporins and septins were suggested to be present in the TZ of locomotory and primary cilia. Here we present data suggesting the presence of nucleoporins, at both nuclear pores and ciliary TZ, in *Tetrahymena *and *Paramecium*. Using the monoclonal 414, specific to FG-containing nucleoporins, and anti-tubulin antibodies, we detected labeling at the distal part of basal bodies and as a spotted pattern around nuclei.

In previous studies on *Tetrahymena*, GFP-tagged septins (Sep1, Sep2 and Sep3) localized to mitochondrial/ER compartments but neither to basal bodies, cilia nor nuclei. *Tetrahymena *cells with knocked out septins display disrupted nuclear (Mac and Mic) membranes, abnormal mitochondria, but unaffected ciliary TZ. Similarly, in *Paramecium*, silencing of the *SEP2 *gene revealed affected nuclear membranes and mitochondria but normal TZ, and the mAb 414 and DAPI showed a normal labeling of the ciliary base, but patches of missing nuclear pores in the macronucleus.

Our studies support the conclusion that some nucleoporins are present in both nuclear pores and ciliary TZ. In addition, the nuclear pore proteins seem to interact with septins, yet the lack of effect of septin silencing on the localization of the mAb 414 in the ciliary TZ suggests that nucleoporins may have different binding partners in nuclear pores and ciliary TZ.

